# Intestinal Ultrasound Combined with Blood Inflammatory Markers Is a More Efficient Tool in Evaluating Severity of Crohn's Disease: A Pilot Study

**DOI:** 10.1155/2023/2173396

**Published:** 2023-11-08

**Authors:** Huaying Fang, Jie Liu, Kai Qian, Xuemei Xu, Zhaolong Li, Li Xie, Menghan Sun, Song Wang, Jiaqin Xu, Chaolan Lv, Bo Wang, Weiyong Liu, Gengqing Song, Yue Yu

**Affiliations:** ^1^Department of Gastroenterology, The First Affiliated Hospital of USTC, Division of Life Sciences and Medicine, University of Science and Technology of China, Hefei, Anhui 230001, China; ^2^Medical Imaging Center, The First Affiliated Hospital of Anhui Medical University North District, Hefei, Anhui 230011, China; ^3^Department of Endoscopy, Shanghai Jiao Tong University Affiliated Sixth People's Hospital, Shanghai 200235, China; ^4^Department of Ultrasound Medicine, The First Affiliated Hospital of USTC, Division of Life Sciences and Medicine, University of Science and Technology of China, Hefei, Anhui 230001, China; ^5^Division of Gastroenterology and Hepatology, MetroHealth Medical Center, Case Western Reserve University, Cleveland, OH 44109, USA

## Abstract

**Background and Aims:**

Intestinal ultrasound (IUS) is considered a nonirradiating, noninvasive, well-tolerated, and valuable tool for objectively assessing Crohn's disease (CD) activity. However, there is no widely accepted intestinal ultrasound scoring system. This study is aimed at evaluating the efficacy of IUS key parameters, the International Bowel Ultrasound Activity Score (IBUS-SAS), and IBUS-SAS combined with blood inflammatory markers in assessing CD activity.

**Methods:**

40 CD patients were reviewed in this retrospective study and were divided into the moderate-severe group (*n* = 25) and nonmoderate-severe group (*n* = 15) based on a simplified endoscopic score of Crohn's disease (SES-CD). Double-balloon enteroscopy/colonoscopy were reviewed by three gastroenterologists. A transabdominal ultrasound was performed by two ultrasound specialists. Blood inflammatory markers were measured from morning samples.

**Results:**

In evaluating moderate to severe CD patients, (1) IBUS-SAS had a good predictive effect with an area-under-the-curve (AUC) of 0.855 (*P* < 0.001); (2) IUS key parameters (including BWT, CDS, BWS, and I-fat) yielded good predictive effects with AUC of 0.811, 0.731, 0.724, and 0.747, respectively (*P* < 0.001); (3) blood inflammatory markers (including ESR, CRP, PLR, MLR, and NLR) also had good predictive effects with AUC of 0.771, 0.837, 0.728, 0.743, and 0.775, respectively (*P* < 0.001); (4) IBUS-SAS combined with ESR and CRP exerted the best predictive effect with the highest AUC of 0.912 (95% CI: 0.823-1.000), and the sensitivity and specificity were 88.0% and 80.0%, respectively (*P* < 0.001).

**Conclusion:**

IBUS-SAS combined with ESR and CRP is a more efficient tool than IBUS-SAS alone or inflammatory markers alone in evaluating CD patients with moderate to severe disease activity.

## 1. Introduction

Crohn's disease (CD) is a chronic, progressive, disabling inflammatory disease of the gastrointestinal tract which can lead to organ damage and impair quality of life. It is a long-course disease with alternating remission and recurrence [1]. Given the disconnection between patient symptoms and disease progression, easily tolerated, objective and accurate tools are needed to assess and monitor CD activity to guide clinical management [2]. A “treat-to-target” strategy based on disease activity and severity and response to treatment with close monitoring of intestinal inflammation are recommended for better long-term clinical outcomes [1, 3]. Endoscopy is considered the gold standard for the assessment of CD activity. Computed tomography enterography (CTE) and magnetic resonance enterography (MRE) are the current standard for assessing the small bowel and complications in CD and have been proposed as alternative procedures to endoscopy in the evaluation of CD activity [4, 5]. As that endoscopy, CTE, and MRE are invasive, time-consuming, and expensive procedures and unappealing to patients, noninvasive tools for assessment and monitoring CD activity are strongly needed.

Intestinal ultrasound (IUS) has several advantages of being well-tolerated, noninvasive, easy repeatability, lack of complex bowel preparation, ionizing radiation, and cost-effective [6]; it is favored by clinicians and patients. Compared to endoscopy, CTE, and MRE, IUS has been shown to have a similar level of accuracy in assessing and monitoring disease activity and severity of CD [6, 7]. In addition, IUS has more advantages than MRE in detecting colonic disease [8]. It can be performed at the point of care and therefore allows for real-time clinical decision-making [7, 9]. Recent ECCO-ESGAR guidelines [10] recommend IUS as the first-line modality for small bowel disease assessment in newly diagnosed CD patients. However, there is no widely accepted, repeatable, consistent IUS scoring system until the emergence of IBUS-SAS in 2021, which makes the IUS scoring system more standardized [11], but this new scoring system needs a large number of clinical studies to confirm its effectiveness.

Erythrocyte sedimentation rate (ESR) and C-reactive protein (CRP) are general markers of inflammation, but their specificity is low [12]. Thus, the new inflammatory markers which are helpful for assessing CD activity are urgently needed [13]. Neutrophil-to-lymphocyte ratio (NLR), platelet-to-lymphocyte ratio (PLR), and monocyte-to-lymphocyte ratio (MLR) had been previously thought to be associated with inflammation and tumors [14, 15] and considered as predictors of disease severity in ulcerative colitis (UC) [16, 17], and a promising marker in predicting the loss of response to infliximab in UC [18]. However, their utility in assessing inflammation and disease activity of CD has still been disagreed with in some aspects [19–21].

Although IUS or blood inflammatory markers alone have been reported to have good accuracy in assessing and monitoring disease activity and severity of CD/UC [13, 17, 22, 23], few studies have shown intestinal ultrasound improvement under treatment correlated with decreased CRP levels [24]. It was unclear whether the combination of these two methods would have a synergistic effect on the assessment of CD activity.

In this study, we aimed to evaluate the diagnostic efficacy of IUS key parameters (BWT, CDS, BWS, and I-fat), IBUS-SAS, and IBUS-SAS combined with blood inflammatory markers (ESR, CRP, PLR, MLR, and NLR) in assessing disease activity in CD patients.

## 2. Materials and Methods

### 2.1. Subject Selection

This retrospective study was approved by the ethics committee of the First Affiliated Hospital of University of Science and Technology of China (USTC, NO 2022-ky-279). A total of 40 cases from hospitalized patients (28 males and 12 females) were collected in the First Affiliated Hospital of USTC between August 2021 and August 2022. These cases were patients diagnosed with CD based on a combination of standard criteria that included clinical symptoms, physical examination, colonoscopy/double-balloon endoscopy (DBE), imaging (CTE or MRE), and histopathology. In addition, all patients joined this study with written informed consent for the research use of their clinical data. The Helsinki Declaration guidelines were followed.

#### 2.1.1. Inclusion Criteria

(1) According to ECCO-ESGAR Guideline [10], the patients who were diagnosed with CD and completed colonoscopy/DBE, IUS examination, blood routine, ESR, and CRP. All data were collected with a maximum interval of 1 month. (2) Age ≥ 14 years, age ≤ 70 years.

#### 2.1.2. Exclusion Criteria

(1) Patients suffer from local or systemic infection. (2) CD isolated with the upper gastrointestinal tract. (3) Patients treated with colon resection and terminal ileum resection, which may result in altered visceral adiposity and IUS parameters. (4) The most severe intestine in the rectum, (5) BMI ≥ 30 kg/m^2^. (6) Pregnancy. (7) Patients treated with prednisone and azathioprine, which may result in altered blood inflammatory markers

### 2.2. Endoscopy and Evaluation

DBE/colonoscopy was performed by three gastroenterologists who each had >10 years of endoscopic examination experience and were blinded to IUS findings. The CD activity was assessed with colonoscopy/DBE by SES-CD [25]. For the SES-CD, the endoscopic variables were evaluated in 5 predefined ileocolonic segments (rectum, left colon, transverse colon, right colon, and terminal ileum), and the 4 endoscopic variables selected were ulcers, proportion of the surface covered by ulcers, proportion of the surface with any other lesions, and stenosis. Each variable was scored from 0 to 3 in each segment. The SES-CD was defined as follows: inactive (0-2), mild activity (3–6), moderate activity (7–16), and severe activity (>16). CD patients with moderate and severe activity were allocated to the moderate-severe group. CD patients with inactive and mild activity were assigned to the non-moderate-severe group [1].

### 2.3. Intestinal Ultrasound

Studies underwent manual measurement of intestinal structural characteristics with IUS by 2 ultrasound specialists with expertise in inflammatory bowel disease and over 20 years of experience who were blinded to subject identifiers, clinical history, endoscopic findings, and each other's measurements using IUS devices (Mindray Eagus R95) and linear probe (L14-5wu).

#### 2.3.1. Bowel Preparation Was Not Routinely Required

But patients were required 8 h of fasting before the IUS and remained supine during the examinations. First, locating the hepatic flexure and then scanning the ascending colon toward the cecum, identifying the terminal ileum. The examination proceeded to the transverse colon, descending colon, and sigmoid colon [26]. The rectum was excluded from this study.

The most serious intestinal segment was selected to observe, and IBUS-SAS was calculated. IBUS-SAS includes evaluation of bowel wall thickness (BWT), bowel wall stratification (BWS), color Doppler imaging signal (CDS), and inflammatory mesenteric fat (I-fat). In longitudinal and cross-section orientations, BWT measurements were performed, and two measurement values were given in each orientation. Loss of bowel wall stratification was defined as a hypoechoic disruption of the 3 distinct wall layers, that is a normal bowel wall stratification characteristic. Evaluation of color Doppler was performed for IBUS-SAS score assessment with a modified Limberg score, assessing the detectable color Doppler signals/pixels inside and outside the bowel wall. I-fat was defined as a homogeneous, hyperechoic change around a thickened bowel wall. And the International Bowel Ultrasound Activity Score (IBUS-SAS) was calculated as follows [11]:

(1) $$\begin{array}{c} \text{IBUS}-\text{SAS}=4\times \text{BWT}+15\times \text{I}-\text{FAT}+7\times \text{CDS}+4\times \text{BWS}. \end{array}$$

### 2.4. CDAI Score

In current clinical practice, Crohn's Disease Activity Index (*CDAI*) is still the gold clinical standard used to assess the inflammatory activity in *CD* [27]. CDAI was determined before DBE or colonoscopy. CDAI was categorized as follows: inactive disease (<150), mild disease (150–220), moderate disease (220–450), and severe disease (>450).

### 2.5. Blood Inflammatory Markers

Blood inflammatory markers included ESR and CRP, MLR, NLR, and PLR. MLR, NLR, and PLR were calculated according to the results of blood routine examination as follows [14, 15]: NLR: neutrophils-to-lymphocytes ratio; PLR: platelets-to-lymphocytes ratio; MLR: monocytes-to-lymphocytes ratio.

### 2.6. Statistical Analysis

The statistical software SPSS22.0 was used to analyze the data. A value of *P* < 0.05 was considered to indicate statistical significance. The measurement data were expressed by average ± standard deviation. Continuous variables were compared using the independent 2-sample *t*-test and Mann–Whitney *U* test according to the normality of their distribution. Nominal variables were compared using the Chi-square test and Fisher's exact test. Associations between two continuous and between continuous and ordinal variables were assessed using Spearman's rank-order correlation. AUC for ROC analysis was used to analyze diagnostic performance.

## 3. Results

### 3.1. Demographic and Clinical Characteristics of CD Patients

A total of 40 patients with confirmed CD were reviewed, comprising 28 males and 12 females, with a mean age of 32.28 ± 12.97. Based on SES-CD as the standard of reference, they were divided into two groups, one group of 25 cases with moderate-severe activity and another group of 15 cases with non-moderate-severe activity. The baseline demographic and clinical characteristics of patients are presented in Table 1.

The clinical manifestations most frequently reported were diarrhea (*n* = 28, 75.0%) and abdominal pain (*n* = 34, 85.0%). The most common complication was stenosis (*n* = 19, 47.5%), followed by anal fistula (*n* = 15, 37.5%) and fistula (*n* = 7, 17.5%). There were 27 patients (*n* = 27, 67.5%) diagnosed as ileocolonic type (Montreal Classification L3), followed by isolated colonic disease (Montreal Classification L2) (*n* = 5,12.5%), isolated ileal disease (Montreal Classification L1) (*n* = 4, 10.0%), and ileocolonic disease involving upper gastrointestinal (Montreal Classification L3 + L4) (*n* = 4, 10.0%). Most patients were treated with a biological agent (adalimumab 3(7.5%), ustekinumab 5(12.5%), Infliximab 10 (25.0%), and vedolizumab 4(10.0%)). Eleven (27.5%) patients were treated with total enteral nutrition. One (2.5%) patient was treated with surgery; another 3 (7.5%) patients were treated with mesalazine.

### 3.2. Comparison of NLR, MLR, PLR, ESR, CRP, and IBUS-SAS between the Two Groups

There was a significant difference in NLR between the non-moderate-severe activity group and moderate-severe activity group (2.14 ± 1.04 vs 3.67 ± 1.95, *P* = 0.008). Significant differences were also found in MLR (0.29 ± 0.12 vs 0.43 ± 0.16, *P* = 0.008) and PLR (161.91 ± 94.63 vs 246.50 ± 114.00, *P* = 0.021) between the two groups. There were also significant differences in ESR, CRP, and IBUS-SAS between the two groups (*P* < 0.005, *P* < 0.001, and *P* < 0.001, respectively) (Table 2).

### 3.3. Correlation between IBUS-SAS, BWT, CDS, BWS, I-fat, and SES-CD

**Figure 1:**
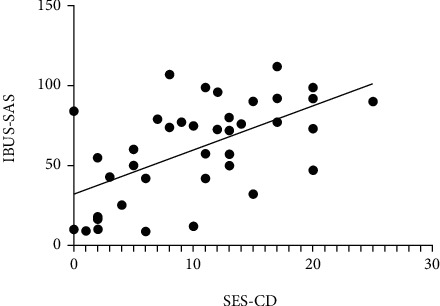
Correlation between IBUS-SAS and SES-CD was strongly positive (*r* = 0.587, *P* < 0.001).

IUS key parameters (BWT, CDS, BWS, and I-fat) and IBUS-SAS score were positively correlated to varying degrees with SES-CD (*P* < 0.05), where the IBUS-SAS score shows the highest correlation (r = 0.587, *P* < 0.001) (Table 3 and Figure 1).

### 3.4. Correlation between IBUS-SAS, BWT, CDS, BWS, I-fat, and CDAI

**Figure 2:**
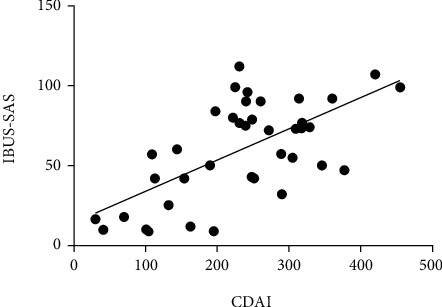
Correlation between IBUS-SAS and CDAI was strongly positive (*r* = 0.640, *P* < 0.001).

A significant correlation was found between IBUS-SAS, BWT, CDS, BWS, I-fat, and CDAI (*P* < 0.001); the highest correlation was found between IBUS-SAS and CDAI (*r* = 0.640, *P* < 0.001), which was a strongly positive correlation (Table 4 and Figure 2).

### 3.5. The Correlation between Inflammatory Markers and SES-CD

There was a positive correlation between blood inflammatory markers (MLR, NLR, PLR, ESR, and CRP) and SES-CD (*P* < 0.01), and the highest correlation was ESR (*r* = 0.656, *P* < 0.001), which was a strongly positive correlation (Table 5).

### 3.6. The Correlations between Inflammatory Markers

The correlation between MLR, PLR, and ESR was considered positively significant, (*r* = 0.360, *P* = 0.023; *r* = 0.488, *P* = 0.001), respectively. The correlation between PLR and CRP was also considered positively significant (*r* = 0.377, *P* = 0.016). However, no statistically significant *correlation* could be determined during CRP, ESR, and NLR (*P* > 0.05) (Table 6).

### 3.7. Evaluation of Intestinal Ultrasound Combined with Blood Inflammatory Markers for CD Activity

**Figure 3:**
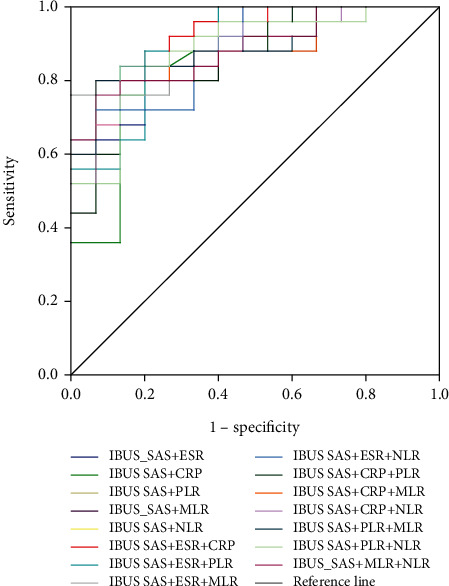
Receiver operating characteristic (ROC) curves of combination between IBUS-SAS and blood inflammatory markers in the diagnosis of moderate-severe activity.

**Figure 4:**
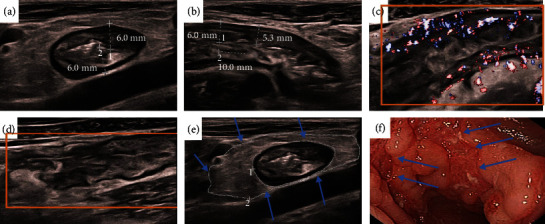
This male CD patient was 18 years old, and he had clinically active CD (CDAI = 360), which was characterized by abdominal pain, diarrhea, and wasting. In our examinations, this was a moderate-severe CD. The SES-CD and IBUS-SAS were 20 and 76 points, respectively. The serious segmental was the sigmoid colon. Application of the segmental activity and severity scores. (a, b) Bowel wall thickness (BWT): In longitudinal and cross-section orientations, BWT measurements were performed, and two measurement values were given in each orientation, [BWT] = [6.0 + 6.0 + 6.0 + 5.3]/4 = 5.8 mm. (c) Blood flow/color Doppler signal [CDS] = 3[short signals]. (d) Bowel wall stratification [BWS] = 3[extensive > 3 cm]. (e) Inflammatory fat [I‐fat] = 2[certain]. (f) Endoscopic image showing a longitudinal ulcer (arrow). The size of ulcer ≥ 4 cm. IBUS‐SAS = 5.8 × 4 + 2 × 15 + 3 × 7 + 3 × 4 = 76.

**Figure 5:**
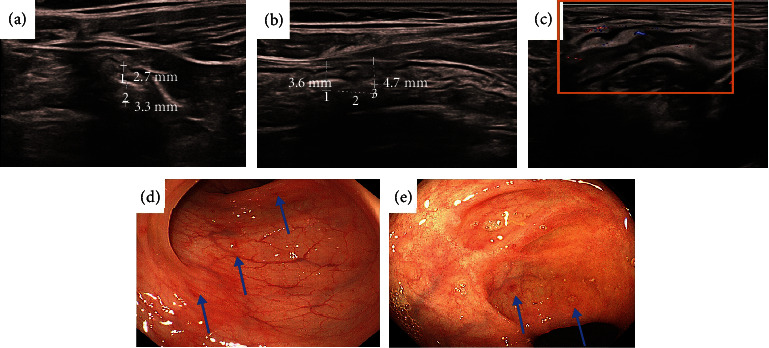
This female CD patient was 20 years old, and the patient had clinically inactive CD (CDAI = 41), which was characterized by diarrhea. In our examinations, this was a non-moderate-severe CD. The SES-CD and IBUS-SAS were 3 and 18.3 points, respectively. The serious segmental was the sigmoid colon. Application of the segmental activity and severity scores. (a, b) Bowel wall thickness (BWT): In longitudinal and cross-section orientations, BWT measurements were performed, and two measurement values were given in each orientation, [BWT] = [2.7 + 3.3 + 3.6 + 4.7]/4 = 3.58 mm. (c) Blood flow/color Doppler signal [CDS] = 1[short signals]. Bowel wall stratification[BWS] = 0, inflammatory fat[I‐fat] = 0 (d), and (e) endoscopic image showing several aphthaes (arrow). IBUS‐SAS = 3.58 × 4 + 0 × 15 + 0 × 7 + 1 × 4 = 18.3.

NLR, MLR, PLR, ESR, CRP, IBUS-SAS, BWS, I-fat, BWT, and CDS have predictive values for moderate-severe CD. When the cut-off value of IBUS-SAS is 56, the AUC is 0.855 (95% CI:0.736-0.974), and the sensitivity and specificity of predicting moderate-severe CD are both 80%. When the cut-off value of ESR is 41 mm/h, the AUC is 0.771 (95% CI:0.621-0.920), and the sensitivity and specificity are 44.0% and 100%, respectively. When the cut-off value of CRP is 7.455 mg/L, the AUC is 0.837 (95% CI:0.693-0.982), and the sensitivity and specificity are 84% and 86.7%, respectively. When IBUS-SAS was combined with PLR, the AUC was 0.864 (95% CI: 0.751-0.977), and the sensitivity and specificity were 80.0% and 86.7%, respectively. When IBUS-SAS was combined with MLR, the AUC was 0.885 (95% CI: 0.783-0.988), and the sensitivity and specificity were 80.8% and 93.3%, respectively. When IBUS-SAS was combined with NLR, the AUC was 0.888 (95% CI: 0.788-0.988), and the sensitivity and specificity were 80.0% and 86.7%, respectively. When IBUS-SAS was combined with ESR, the AUC was 0.888 (95% CI: 0.789-0.987), and the sensitivity and specificity were 88.8% and 73.3%, respectively. When IBUS-SAS was combined with CRP, the AUC was 0.849 (95% CI: 0.722-0.976), and the sensitivity and specificity were 76% and 86.7%, respectively. When IBUS-SAS was combined with ESR and CRP, the AUC showed the highest value of 0.912 (95% CI: 0.823-1.000) than other combinations, and the sensitivity and specificity are 88% and 80%, respectively. Although no significant difference was found, the sensitivity was 80% and 88% for IUS alone and combined with ESR/CRP, respectively (Tables 7 and 8 and Figures 3–5).

## 4. Discussion

ECCO guidelines [1] indicated that the treatment and prognosis of moderate-severe CD were different from those of mild CD and those in remission and that early use of biological agents is preferred for moderate-severe CD patients. This study has identified the more effective diagnostic tool for assessing CD activity to guide clinical treatment. The highlight of this study is to combine IUS and blood inflammatory markers, which are noninvasive and simple monitoring, in order to better distinguish moderate-severe CD for the first time.

IUS has a great advantage in monitoring the activity of CD, but IUS has not been widely used in the clinic because IUS relies on standardized score [28]. A meta-analysis published in 2018 showed that ultrasound activity scores mostly had significant limitations, and none has been adequately validated [29].

It is encouraging that the IBUS-SAS [11], which was a standardized score, was submitted for publication in 2021. The purpose of this study was to examine the validation of IBUS-SAS. In a rigorous attempt to standardize measurement, it endeavored to optimize acquisition and measurement techniques with the aim of limiting uncertainty in the interpretation and grading of individual parameters. IBUS-SAS not only can be used to predict and evaluate the segmental bowel but also for the overall prediction and assessment of diseases. Therefore, the most severe intestinal segments were selected for IUS examination in this study for overall comparison. Unexpectedly, one recent study on IBUS-SAS found that it was not able to accurately correlate endoscopic activity in the terminal ileum in CD in this year [30]. In contrast, we found that the correlation between IBUS-SAS and SES-CD and CDAI was considered strongly significant (*r* = 0.587, 0.640, respectively). In addition, there was a significant difference in IBUS-SAS between moderate-severe and non-moderate-to-severe groups. The ROC curve revealed that a cut-off score of IBUS-SAS was 56, and the AUC was 0.855 for predicting moderate-severe CD activity. Among the key parameters of IBUS-SAS, the reliable measurement of BWT is central to consistent interpretation for the diagnosis and evaluation of CD activities. This study also showed that BWT was positively correlated with SES-CD and CDAI (*r* = 0.472, 0.624, respectively). Compared with other IUS parameters, BWT is a higher correlation than others. Another study confirmed that the sensitivity and specificity of *BWT* > 3 mm in detecting CD inflammatory activity were 100% and 83%, respectively, while the specificity of BWT > 4 mm was 91.6% [31]. Our study found that when BWT was 4.8 mm, it had a good predictive value for moderate-severe CD, and the sensitivity and specificity were 80% and 66.7%, respectively. In addition, previous studies have shown that I-fat, BWS, and CDS were all associated with disease activity [28]. This study again confirmed that these key parameters were closely associated with CD disease activity. I-fat was the most correlated indicator of disease activity after BWT [32]. This study also found that I-fat correlated with the Crohn's *disease activity* index. The correlation between I-fat and SES-CD was 0.509, and the correlation between I-fat and CDAI was 0.345. The AUC of I-fat was 0.747, and the sensitivity and specificity of I-fat for detecting moderate-severe CD activity were 76.0% and 73.0%. Our study confirmed that the IBUS-SAS score, including its key parameters, was a good scoring system for CD activity.

NLR, MLR, and PLR are biomarkers of systemic inflammation, but studies in CD are still not in-depth, and there are still disagreements in some aspects. Eraldemir et al. [19]suggested that PLR and NLR values could evaluate CD activities. However, Bou Jaoude [20] reported that PLR and NLR had no discriminating values in distinguishing mild Crohn's disease from controls, or between mild active and inactive Crohn's disease. Zhang et al. [21] found that both the *accuracy* of NLR and MLR were 86.4% for distinguishing severe CD from mild-moderate CD, but NLR displayed the best AUC of 0.89. Meanwhile, this study identified a significant correlation between NLR, MLR, PLR, and SES-CD. In addition, there was a significant difference in NLR, MLR, and PLR between the two groups, and the ratios in the moderate-severe group were significantly higher than that in non-moderate-severe group. Finally, the ROC curve showed that NLR, MLR, and PLR had clinical predictive value for moderate-severe activity in CD. The ROC curve for NLR had been found to have the best AUC of 0.775 with a suggested optimal cut-off of 2.82 to differentiate moderate-severe CD patients from non-moderate-severe CD (sensitivity 64.0% and specificity 86.7%). Our results suggested all the above blood inflammatory markers could evaluate the CD activity *to a certain extent*.

CRP and ESR have also been shown to be significantly associated with the severity of CD and have been widely used to monitor activity in clinical IBD disease [33]. Our study found that ESR and CRP could be utilized as indicators of moderate-severe inflammation. The ROC curve for ESR had been found to have a better AUC of 0.771. When the cut-off value of the IBUS-SAS score was 56, that of ESR was 41 mm/h, and that of CRP was 7.455 mg/L, they could predict moderate-severe CD. IBUS-SAS combined with CRP or ESR could significantly improve the diagnostic efficiency of predicting moderate-severe CD. The AUC was calculated from 0.885 to 0.912.

However, this study also had some limitations. Firstly, it was a single-center retrospective study with a small sample size, which may potentially impact the generalizability of the findings. Secondly, some patients received different treatments at the time of enrollment, which may affect the results of peripheral blood inflammatory indicators. Thirdly, the male-female ratio of the CD patients was biased (28 males and 12 females); Finally, the study did not include the observation of CD complications such as stenosis, fistula, and abscess, which could also predict disease activity.

In conclusion, IUS key parameters, IBUS-SAS scores, and blood inflammatory markers such as NLR, MLR, PLR, ESR, and ESR correlated with CD *disease activity*. IUS key parameters, IBUS-SAS scores, and blood inflammatory markers (NLR, MLR, PLR, ESR, and CRP) could effectively predict moderate-severe CD activity. IBUS-SAS combined with ESR and CRP can achieve better predictive efficacy in evaluating the severity of CD. Our data need to be confirmed and validated in further large-scale multicenter studies.

## Figures and Tables

**Table 1:** Baseline demographic and clinical characteristics of CD patients.

Characteristics	*n* = 40 (%)
Sex	
Men (*n*, %)	28 (70.0%)
Women (*n*, %)	12 (30.0%)
Median age (*x* ± *s*)	32.28 ± 12.97
Symptoms	
Abdominal pain (*n*, %)	34 (85.0%)
Diarrhea (*n*, %)	28 (70.0%)
Wasting (*n*, %)	17 (42.5%)
Gastrointestinal bleeding (*n*, %)	10 (25.0%)
Extraintestinal manifestations (*n*, %)	8 (20.0%)
Comorbidities and complications	
Fistula (*n*, %)	7 (17.5%)
Stricture (*n*, %)	19 (47.5%)
Anal fistula (*n*, %)	15 (37.5%)
Abdominal abscess (*n*, %)	1 (2.5%)
Abdominal abscess	
L1, ileal (*n*, %)	4 (10.0%)
L2, colonic (*n*, %)	5 (12.5%)
L3, ileocolonic (*n*, %)	27 (67.5%)
L3 + L4 ileocolonic + upper gastrointestinal (*n*, %)	4 (10.0%)
Treatment	
Mesalazine (*n*, %)	3 (7.5%)
Adalimumab (*n*, %)	5 (12.5%)
Ustekinumab (*n*, %)	4 (10.0%)
Infliximab (*n*, %)	10 (25.0%)
Vedolizumab (*n*, %)	4 (10.0%)
Enteral nutrition (*n*, %)	11 (27.5%)
Surgical (*n*, %)	1 (2.5%)

**Table 2:** Comparison of sex, years, NLR, MLR, PLR, ESR, CRP, and IBUS-SAS between the two groups.

	Non-moderate-severe (*n* = 15, %)	Moderate-to-severe (*n* = 25, %)	*P*
Sex			0.154
Men	7 (46.7)	5 (20.0)	
Women	8 (53.3)	20 (80.0)	
Years	38.00 ± 12.91	28.84 ± 12.27	0.031
NLR	2.14 ± 1.04	3.67 ± 1.95	0.008
MLR	0.29 ± 0.12	0.43 ± 0.16	0.008
PLR	161.91 ± 94.63	246.50 ± 114.00	0.021
ESR (range, median)	(2-38, 8)	(4-104, 29)	0.005^∗^
CRP (range, median)	(3-84, 3.16)	(3.13-86.6, 11.9)	<0.001^∗^
IBUS-SAS (range, median)	(8-84, 42)	(12-112, 77)	<0.001^∗^

^∗^ *p* < 0.050.

**Table 3:** Correlation between IBUS-SAS, BWT, CDS, BWS, I-fat, and SES-CD.

	SES-CD		
	*r*	*P*	Correlation level
BWT	0.472	0.002	Moderate
CDS	0.384	0.014	Moderate
BWS	0.382	0.015	Moderate
I-fat	0.509	0.001	Strong
IBUS-SAS	0.587	<0.001	Strong

**Table 4:** Correlation between IBUS-SAS, BWT, CDS, BWS, I-fat, and CDAI.

	CDAI	
	*r*	*P*
IBUS-SAS	0.640	<0.001
BWT	0.624	<0.001
CDS	0.576	<0.001
BWS	0.571	<0.001
I-fat	0.345	<0.001

**Table 5:** Correlation between inflammatory markers and SES-CD.

	SES-CD	
	*r*	*P*
NLR	0.408	0.009
MLR	0.482	0.002
PLR	0.425	0.006
ESR	0.656	<0.001
CRP	0.556	<0.001

**Table 6:** Correlation between NLR, MLR, PLR, ESR, and CRP.

	ESR		CRP	
	*r*	*P*	*r*	*P*
NLR	0.306	0.055	0.250	0.119
MLR	0.360	0.023	0.222	0.168
PLR	0.488	0.001	0.377	0.016

**Table 7:** Evaluation of detection of IUS parameters, IBUS-SAS, ESR, CRP, PLR, MLR, and NLR in the diagnosis of moderate to severe activity.

	Cut-off value	Sensitivity (%)	Specificity (%)	AUC	95% CI	*P*
IBUS-SAS	56.0	80.0	80.0	0.855	0.736-0.974	<0.001
BWT	4.8	80.0	66.7	0.811	0.675-0.946	0.001
CDS	1.5	76.0	66.7	0.731	0.559-0.902	0.016
BWS	1.5	80.0	60.0	0.724	0.547-0.901	0.019
I-fat	1.0	76.0	73.3	0.747	0.583-0.910	0.010
ESR	41.0	44.0	100.0	0.771	0.621-0.920	0.005
CRP	7.455	84.0	86.7	0.837	0.693-0.982	<0.001
PLR	237.605	56.0	86.7	0.728	0.567-0.889	0.017
MLR	0.355	64.0	80.0	0.743	0.581-0.904	0.011
NLR	2.82	64.0	86.7	0.775	0.629-0.921	0.004

**Table 8:** Evaluation of combined detection of IBUS-SAS and blood inflammatory markers in the diagnosis of moderate to severe activity.

	Cut-off value	Sensitivity (%)	Specificity (%)	AUC	95% CI	*P*
IBUS-SAS + ESR	—	88.0	73.3	0.888	0.789-0.987	<0.001
IBUS-SAS + CRP	—	76.0	86.7	0.849	0.722-0.976	<0.001
IBUS-SAS + PLR	—	80.0	86.7	0.864	0.751-0.977	<0.001
IBUS-SAS + MLR	—	80.0	93.3	0.885	0.783-0.988	<0.001
IBUS-SAS + NLR	—	80.0	86.7	0.888	0.788-0.988	<0.001
IBUS-SAS + ESR + CRP	—	88.0	80.0	0.912	0.823-1.000	<0.001
IBUS-SAS + ESR + PLR	—	88.0	80.0	0.893	0.793-0.994	<0.001
IBUS-SAS + ESR + MLR	—	76.0	100	0.907	0.819-0.995	<0.001
IBUS-SAS + ESR + NLR	—	72.0	93.3	0.888	0.789-0.987	<0.001
IBUS-SAS + CRP + PLR	—	80.0	86.7	0.864	0.751-0.977	<0.001
IBUS-SAS + CRP + MLR	—	80.0	93.3	0.883	0.779-0.986	<0.001
IBUS-SAS + CRP + NLR	—	84.0	86.7	0.888	0.787-0.989	<0.001
IBUS-SAS + PLR + MLR	—	80.0	93.3	0.891	0.791-0.990	<0.001
IBUS-SAS + PLR + NLR	—	84.0	86.7	0.885	0.779-0.991	<0.001
IBUS-SAS + MLR + NLR	—	76.0	93.3	0.885	0.785-0.985	<0.001

## Data Availability

All data generated or analyzed during this study are included in this published article. The data used to support the findings of this study are available from the corresponding authors upon request. Data were collected by authorized researchers.
